# Healthcare worker perceptions of the implementation context surrounding an infection prevention intervention in a Zambian neonatal intensive care unit

**DOI:** 10.1186/s12887-020-02323-2

**Published:** 2020-09-10

**Authors:** Carter Cowden, Lawrence Mwananyanda, Davidson H. Hamer, Susan E. Coffin, Monica L. Kapasa, Sylvia Machona, Julia E. Szymczak

**Affiliations:** 1 Center for Pediatric Clinical Effectiveness and Division of Infectious Diseases Children’s Hospital of Philadelphia, Philadelphia, PA USA; 2 Right to Care Zambia, Lusaka, Zambia; 3 Department of Global Health, Boston University School of Public Health and Section of Infectious Diseases, Department of Medicine, Boston University School of Medicine, Boston, MA USA; 4 Department of Pediatrics, Perelman School of Medicine, University of Pennsylvania, Philadelphia, PA USA; 5 Department of Pediatrics and Child Health, University Teaching Hospital, Lusaka, Zambia; 6 Department of Biostatistics, Epidemiology and Informatics, Perelman School of Medicine, University of Pennsylvania, Blockley Hall Room 708, 423 Guardian Drive, Philadelphia, PA 19104-4302 USA

**Keywords:** Infection prevention, Pediatrics, Implementation science, Qualitative methodology, Zambia, Intensive care, Bloodstream infections

## Abstract

**Background:**

Infants in the neonatal intensive care unit (NICU) are particularly susceptible to healthcare-associated infections (HAIs). NICUs in low- and middle income countries face additional challenges to HAI prevention. There is a need to better understand the role of the implementation context surrounding infection prevention interventions in low- and middle income countries.

**Aim:**

The aim of this study was to identify NICU healthcare worker perceptions of an intervention to reduce bloodstream infections in a large Zambian NICU.

**Methods:**

Semi-structured interviews were conducted with NICU staff during a prospective cohort study examining the impact of an infection prevention bundle on bloodstream infections. Interviews were analyzed using an integrated approach, combining inductive theme generation with an application of the Consolidated Framework for Implementation Research (CFIR).

**Results:**

Interviews were conducted with 17 NICU staff (5 physicians and 12 nurses). Respondents believed the bundle elements were easy to use, well-designed and facilitated improved performance. Four organizational characteristics that facilitated HAI transmission were identified – (1) lack of NICU admission protocols; (2) physical crowding; (3) understaffing; and (4) equipment shortages. Respondents suggested that NICU resource constraints reflected a societal ethos that devalued the medical care of infants. Despite the challenges, respondents were highly motivated to prevent HAIs and believed this was an achievable goal. They enthusiastically welcomed the bundle but expressed serious concern about sustainability following the study.

**Conclusions:**

By eliciting healthcare worker perceptions about the context surrounding an infection prevention intervention, our study identified key organizational and societal factors to inform implementation strategies to achieve sustained improvement.

## Background

Healthcare-associated infections (HAI) cause substantial morbidity and mortality worldwide, and patients hospitalized in low and middle income countries suffer from a greater prevalence of HAIs than those in high-income countries [1, 2]. Patients in neonatal intensive care units (NICUs) are especially susceptible to HAIs and therefore are at higher risk for longer hospital stay and death [3]. Most research on neonatal HAIs and infection prevention has been conducted in high-income countries; less is known about successful interventions in low and middle income countries [4]. HAI transmission is shaped by local contexts surrounding the delivery of care and healthcare worker perceptions of infection prevention, and these can differ markedly across settings [5–7].

Infection prevention initiatives in low and middle income countries are becoming more prevalent through partnerships with high-income countries. While these partnerships can provide much-needed resources, they face challenges and often succumb to implementation pitfalls such as differing partner interests, priorities, ownership, commitment, coordination and communication [6, 8, 9]. There is a critical need to understand how the implementation context influences the success of infection prevention interventions that leverage international partnerships [10]. This inquiry can maximize intervention effectiveness, promote sustainability and minimize pitfalls that arise when interventions are misaligned with the systems in which they are implemented [11, 12].

The Consolidated Framework for Implementation Research (CFIR) has been widely used to guide inquiry into the context surrounding multicomponent healthcare delivery interventions from the perspective of key stakeholders [13–16]. The five major CFIR domains are (1) intervention characteristics, (2) outer setting (i.e. the social, economic and political context surrounding the organization), (3) inner setting (i.e. features of the organization), (4) characteristics of individuals involved in the intervention (i.e. healthcare workers), and (5) the implementation process. Information generated by examining each domain from the perspective of stakeholders affected by an intervention can enable the development of implementation strategies to achieve spread and sustained improvement.

Our team recently completed a large observational cohort study to measure the impact of a low-cost infection prevention bundle on rates of neonatal hospital-acquired bloodstream infections (BSIs) as well as overall mortality in a large Zambian NICU [17]. Implementation of the bundle led to an observed reduction in rates of suspected sepsis, BSIs and death among neonates in the NICU. To identify staff perceptions of the bundle, implementation process and factors that facilitate transmission of HAI, we collected qualitative interview data with NICU staff during the study implementation phase.

## Methods

### Design, setting and sample

Semi-structured interviews were conducted with doctors and nurses working in a NICU at a large public hospital in Lusaka, Zambia, that serves as the referral center for a catchment area of 2–4 million. The unit had roughly 3500 admissions per year, with 75% admitted from the in-hospital labor and delivery (L&D) ward. The remaining 25% of babies were delivered outside the hospital at health centers and private hospitals around Lusaka that do not have high level newborn care capacity. Overcrowding and understaffing were chronic problems. The NICU staff had a large caseload, often having 75 patients with only a 60 patient capacity and a patient to clinician ratio of 20:1. HAIs were common (22% during baseline period). Overall mortality before the intervention ranged from 35 to 50% [18].

Two researchers (a medical sociologist and research associate) from the United States (U.S.)-based study team visited the NICU for 3 weeks to collect qualitative data two months after bundle implementation. The bundle consisted of:

1. Workshop to train staff on the importance of hand hygiene and equipment sterilization,
2. Production of an alcohol-based hand rub using locally-sourced ingredients and placement of 20 dispensers around the unit
3. Bathing of all babies with both a weight of ≥1.5 kg and corrected gestational age of ≥32 weeks using 2% chlorhexidine gluconate (CHG) solution
4. Text message reminders sent daily about infection prevention
5. Enhanced environmental cleaning

All healthcare workers that participated in the implementation of the bundle were eligible for participation. Respondents were recruited in person by the two researchers with assistance from NICU physician and nurse leaders, who provided us with the names and roles of staff. The researchers announced their presence and purpose at a meeting with NICU staff at the beginning of the three-week period, inviting staff to participate in an interview. They visited the NICU each day over the 3 week period, conducting interviews that were scheduled in the introductory staff meeting and approaching others as they began or ended their work shift. They recruited staff until thematic saturation was achieved. The researchers monitored for saturation by keeping an ongoing data collection memo which identified emergent themes [19]. Memos were reviewed at the conclusion of each day.

### Data collection

All interviews were conducted on-site, usually just before or after a respondent’s work shift. Interviews took place in a quiet, semi-private corner of the NICU. The study aims were explained to each respondent, and all questions were answered before obtaining verbal consent. The interviews were conducted using a guide that included a series of open-ended questions intended to elicit staff perceptions of bundle elements (i.e. hand rub, CHG bathing, text message reminders, infection prevention training workshop), as well as infection prevention in the NICU generally. Example open-ended questions included: What are your opinions about the chlorhexidine bathing protocol that was implemented in the NICU? What are your thoughts about why NICU patients get bloodstream infections while getting care in this hospital? What gets in the way of preventing hospital-acquired infections in this NICU? How did it feel to be participating in this study? The complete interview guide is included as an Additional File. Interviews were audio recorded and transcribed.

### Data analysis

Transcripts were uploaded to QSR NVivo 11 software for management and coding. We utilized an integrated analytic approach that combined an application of CFIR constructs to the data with the identification of salient, repeated themes that emerged from the data [20]. First, we created the codebook. The researchers read through the transcripts and identified themes that repeatedly arose while examining the content of the data collection memos. Inductive themes were clearly defined and created as codes. At this stage we determined that incorporating an implementation science framework to organize our inductive findings would both sensitize us to important contextual mechanisms present in our data and engage with well-established analytic taxonomy, terminology and defintions [13]. To complete our codebook, we created codes based on the five major CFIR domains, using the framework’s constructs to define each code [16]. Second, the research associate read the interviews line-by-line and applied codes to passages of text. The medical sociologist double-coded every third interview and intercoder agreement was assessed in NVivo, which was consistently > 90%.

The Boston University Institutional Review Board and the Excellence in Research Ethics and Science (ERES) Converge ethics committee based in Lusaka, Zambia granted approval for this study. Other organizations that approved the research were the National Health Research Authority (the Ministry of Health) and the Department of Pediatrics at the study hospital.

## Results

All 17 NICU healthcare workers who were invited participated in an interview. Of these respondents, 13 were female, 12 were nurses and 5 were physicians, and they had been working a median of 30 months in the NICU (IQR: 11.4–63 months). The participants had a range of experience in this hospital’s NICU, with some interviewees having less than a year of experience, and others having worked there for over a decade.

### Domain 1: intervention characteristics

Respondents were largely enthusiastic about the intervention and believed the bundle was reducing BSIs and mortality. Doctors and nurses expressed enthusiasm for CHG bathing, especially because it was widely noted that prior to the study, regular bathing was not otherwise occurring:

*Considering that most babies are not bathed in water and soap when they come here unless they are very, very dirty and we actually do have the time, the study has been helpful in reducing the amount of bacteria that’s on their skin and that reduce the amount of bacteria going around as you handle other babies*. [Nurse 8].

Alcohol hand rub was perceived as time-saving, easy to use and helpful in the face of water shortages:

*I like it. Because in the night you find that maybe the water, the water goes away. ‘Cause these [hand washing] buckets aren’t always filled. Even when water is available the buckets don’t always have water, so you use it.* [Nurse 2].

Some respondents did not like the texture or smell of the hand rub and believed it led to excessive hand dryness:

*I don’t like this strong alcohol smell. Depending on the day, sometimes, I get this nasal congestion, all the time. I’m sneezing, I’m coughing, it’s irritating for me. But I use it.* [Doctor A].

The placement of dispensers was also described as a problem and respondents suggested hand rub should be located closer to the point of care:

*If you go into the ward, so you find one, is put in that corner, and you have all the babies on this side. So you are seeing this baby. So if you are seeing this baby, you have to walk to that corner to go and hand rub and – this is the same thing, waste of time, going there, might as well see one, two babies, quickly, then I go and do hand rub. So I think that the positioning also maybe puts a challenge on people. Maybe we need containers so each – wherever each baby is, there is a container for the hand rub.’* [Doctor C].

The text messages reminders, which included brief messages reinforcing infection prevention techniques interspersed with neonatal care best practices, were seen as providing valuable information in an efficient format:

*They are good and irritating [laughter]. It’s irritating in the sense that your phone is always buzzing. You’re maybe waiting for that one text message, and it’s not the one from the person you are waiting for [laughter]. But you read it anyway.* [Doctor B].

### Domain 2: outer setting

In making sense of the unit’s infection prevention challenges, physicians suggested that a root cause was societal attitudes toward newborns, caused by historically high neonatal mortality:

*There’s a belief that if a baby is preemie or when the baby is born, the family won’t get too excited until they get to a certain stage. They don’t even name that child.* [Doctor B].

There was a belief that mothers could have another baby to ‘replace’ an infant who died, and therefore intensive neonatal care was not thought to be a priority:

*One of the mentalities that have been here for a very long time is that children are neglected. They’re treated as small adults. They really don’t pay much attention to the children, especially to the newborn. People just say ‘oh, it’s a newborn, probably have another one.’ People just feel really, they’d rather pay attention to the adults.* [Doctor D].

Physicians, especially, spoke about a generalized sense of cynicism within the clinical community that premature babies could survive:

*Because they don’t look at that child as it could potentially survive, and that’s the challenge we have with preemies …*. M*aybe that’s why there isn’t so much support to the preemie program, but we want to raise awareness … So I know there are preemies who are here that they are older now, working. They could tell their stories … and people see, ‘Okay, fine. You can be less than a kilo and survive.’* [Doctor A].

It was suggested that this attitude led to a lack of public awareness about the NICU and subsequent devaluation by hospital administrators. Respondents believed that resource allocation reflected these priorities. The adult ICU was perceived to be staffed adequately, and received necessary equipment when requested. Respondents described occurrences of equipment being removed from the NICU and taken to adult units:

*So they’ll look at you and think, you are not necessary. So eventually, because of political interference, the x-ray machine was taken from main ICU to a local clinic. So they had to grab – it was management’s decision to get the x-ray machine from here, and take it to main ICU to be stationed there. So we argued, what’s the difference? We’re also an ICU. If people want to use it, they can come and get it from here and go and do x-rays. They say, ‘no, this is a management decision. Get that machine from there. Let it be stationed in main ICU.’* [Doctor C].

### Domain 3: inner setting

Respondents identified four organizational features that facilitated infection transmission. First, the admission of babies to the unit was an uncontrolled process in which contamination was likely to occur. Of specific concern was the hospital’s L&D ward, which was thought to be the first exposure to infection for neonates:

*You have to start from the labor ward. I’ve gone there to receive a baby, they call and say ‘we have a mother with complications, please come.’ So you go there, and you don’t have everything that you need right there and then. For example, the suctioning tube, somebody would have been suctioning a baby there. And they leave whatever suctioning tube they used hanging, without discarding it. So if I’m not careful, I don’t change the suctioning tube, I’ll cause sepsis. I don’t know how many midwives actually change that suctioning tube … Labor ward has got a very small bed capacity – 16. And they have 60, 70, 80 deliveries per day. So they have 16 bed spaces. So some deliver in the corridors, on the floor. So all that is a very high risk factor of sepsis. So there’s a lot of things that go on there. Then, the baby comes to us. When the baby comes to us, sometimes we have so many admissions in a very little time, so sometimes it’s difficult to actually change the lid on the resuscitator. Sometimes, linen is not available on time. Especially on the weekend, we run out of linen. So we find new babies in old bedding and the resuscitator is used by two, three, four babies.* –[Doctor 2].

Respondents suggested that conditions in the L&D ward were unhygienic, in large part because the ward operated well above capacity, leading to cramped conditions and many opportunities for contamination. After birth, babies were carried upstairs to the NICU directly without bathing:

*We receive the baby and they’ve told us ‘we’ve suctioned.’ If they haven’t followed the infection prevention techniques from there when suctioning, it means the baby will have come with an infection from there...We are supposed to bathe the baby but the issue is manpower, so we don’t always do it. But there are those that you really have to do as they come from labor and delivery covered with vernix and meconium. So those we bathe.* [Nurse 4].

Many respondents attributed some of the infections seen in the NICU to transmission that happened in the L&D ward.

Second, respondents believed the NICU admitted too many patients, a number of whom did not require critical care, leading to physical crowding:

*Half of them don’t need to be here. The big babies – so labor ward will send for anything. They’ll send an infant of a diabetic mother who is stable. They’ll send a baby been delivered in elective or medical cesarean section, they’re hypothermic – rather than warm the baby they’ll send them to the NICU. So they’ll send everyone, you know. And then because we have infection rate is very high here, and we don’t know exactly what transpired to that mother, in terms of the risk factors, we will keep these babies here until we have investigated and make sure everything is okay before we send them out, okay? So that puts a lot of work load, in terms of babies that don’t need to be here on the ward, and be with the mother there. And there’s no space...Yesterday I was doing rounds I think I had 28 babies. Half of them were stable, just need to be with the mothers to feed and go home. There are probably six or seven that need to be here in terms of critical, because they need oxygen and things like that. But because of those logistics, everybody ends up being here, even the big babies end up staying here for a long time, end up getting infection along the way. And they’ll probably die, even if when they came in they were very stable.* [Doctor E].

As a result of this physical crowding, babies were sometimes placed two or more to a bed, and were not separated by illness severity:

*Sometimes you are forced to put two babies in one cot because you don’t have enough beds. We run out of cots when the ward is very full. So you have a lot of admissions, but you still need to admit this baby. So you end up putting two babies in one cot instead of one.* [Nurse 7].

Patients with suspected infection were not separated or quarantined. NICU staff described tending to babies one right after the other, since there was no space or physical cue to perform hand hygiene after each patient:

*Because one of the challenges here with the layout of the unit is just how close the babies are together. So there’s not that – like as you’re saying, when you’re going from baby to baby, wash your hands in between, but there’s like no physical space.* [Doctor A].

*It is hard because you are handling one baby, and the next baby starts crying. They’re right there, next to you. So immediately you want to hold them, but you were just holding the other baby. So you don’t think to leave and wash your hands. So it is a bit of a challenge.* [Nurse 12].

Third, all respondents perceived that the NICU was understaffed for the number of patients. Nurses, in particular, expressed frustration with the nurse-to-patient ratio:

*We are understaffed. There are a lot of babies. According to my training I’m supposed to see four babies per day. But sometimes as you can see I’m here alone. Sometimes we don’t really have students and there are maybe 30 or 40 babies.* [Nurse 11].

They recognized that there were certain basic nursing practices that they should be providing (like bathing) but felt they did not have the time to perform them, especially when there were life-saving procedures to be completed:

*It [bathing] should be part of our work, but I think looking at the workload, we don’t do it. You’d rather do the nursing care – it’s part of nursing care. Let me not remove it from the nursing care. It is also part of the nursing care, but it’s not them. I don’t know. The place is just overwhelming with work. So you think bathing, I’d rather not do it. I would rather do life-saving measures, and then the bathing, but it’s actually very important.* [Nurse 4].

Nursing students were present to help with the workload, but lacked infection prevention training and frequently touched babies without proper technique:

*Here, the nursing students all have access, everyone touches, everyone does what they want. The students take vitals and if you watch you’ll see they move one stethoscope between five, ten babies. They don’t swab it. You can almost see the Klebsiella being transmitted. It’s the tiny things. But when I’ve tried to say something to stop it, I feel like I’m being controversial, I’m fighting the system. This nurse has five students who are helping her and she needs them because she is assigned 35 preemies to watch. So if you say something you come off as “the doctor doesn’t want the nurse to get help” and I rely on the nurses so I can’t jeopardize our relationship by correcting her students.* [Doctor C].

Speaking up to correct breaches in infection prevention protocol to this group of personnel in the context of understaffing, as this physician describes, is not a straightforward task.

Fourth, equipment and supply limitations was described as a constant challenge. Limited numbers of portable suction machines meant that these devices were often used on multiple babies without proper cleaning and disinfection between each use:

*The other reason is the suctioning machines that we use we have – Okay right now at least we’ve been given about one in each room. Previously we only had about two for the whole ward. Then you have to suction the babies. But even the one that we have it’s just like one in each room. So at times you – Mostly you just have to make sure that the suctioning machine is clean before you use it but at times it becomes a challenge. If maybe the person that was using it previously didn’t make sure it was clean enough. Then there’s an emergency and somebody just gets the suction machine and uses it.* [Nurse 2].

### Domain 4: characteristics of those involved in implementation

Despite the aforementioned challenges we found that all respondents believed it was possible to reduce HAIs in the NICU and that this was an important goal:

*We had an attack of Klebsiella, earlier this year..And you’d see a baby change within hours from pink to gray. The baby just starts bleeding from the nose, from the mouth, and you lose babies like that. So we know how scary it is to have severe infections. So people are trying really hard so that we do not have Klebsiella.* [Nurse 1].

*There’s one thing I like about them [the staff here], they won’t wait. They’ll try everything. They don’t just give up. At other places they complain the whole day. Here, they don’t sit. Because they know we can make a difference [to prevent infection] but we just need to convince the right people to back us up*. [Doctor B].

They expressed motivation to implement infection prevention measures and to be accountable for their role in causing HAIs. However, this accountability-taking and motivation was tempered by the realities of resource limitations. Respondents described a sense of weariness and guilt that arose from knowing what one should do to prevent infection with what was possible given the daily challenges of providing care in a resource-limited setting:

*There are so many things I would want to do for our patients, but we are unable to do because you just can’t do it on your own. You’re so few, and you’re so stressed out with the workload that you have. I go home, I’m so exhausted, I can’t do anything at home because I’m so tired. Sometimes I even feel lazy to bathe my own when I get home, you know? My own kids, I can’t even bathe them. I ask somebody else to bathe them. Even myself when I get home.* [Nurse 9].

*You always have a guilt. On each and every patient that dies. You have a percentage of guilt in you. There is something that I might have done wrong that has caused this. But the other thing is also whereby you know your hands have been held. You can’t do – you tried your best. It’s a balance between the two. Between guilt and trying your best.* [Doctor A].

While the motivation they expressed in the overall goal of HAI prevention was high, respondents expressed some doubt in their own capabilities, given resource limitations, to reliably execute infection prevention measures.

### Domain 5: implementation process

In reflecting on the implementation process surrounding the study, respondents described three key issues. First, obtaining consent from mothers to bathe the babies with CHG solution was routinely mentioned by doctors and nurses as a barrier to timely completion of the task:

*When the study started I was very excited, I thought it [CHG bathing] would be done on every baby. But I have found out with time that it’s not really with every baby. They have to get consent since it is a study. Maybe when something comes out of it they come to the conclusion that it can be done on everybody routinely. It will help a lot.* [Doctor B].

A number of respondents expressed some impatience about making CHG bathing a part of routine practice, since many were aware of research from other settings demonstrating efficacy:

*If the results are known and the results are good, I was wondering why it [CHG bathing] should be studied. It should just be an implementation program.* [Nurse 3].

Second, respondents suggested that the education and messaging about the bundle should extend beyond doctors and nurses to students, maids and nutrition staff:

*I feel it can be prevented. I feel it can but if we fight this infection I think everyone in the unit should be incorporated. They should maybe do some kind of capacity building or awareness where everyone knows and not just the nurses. We have other kinds, like the maids who clean, the nutrition demonstrators, they should be taught also, the importance of infection prevention since we are together in this.* [Nurse 3].

Third, all respondents expressed fears about the sustainability of bundle elements after study completion:

*The thing is, whatever you start, you should always look at the sustainability of it. Will the chlorohexidine be always present? I guarantee you that within six months, there will be no chlorohexidine present. It will be. So better choose a method, look for a method that can be consistent with it. Because this won’t be available. Someone has donated it. So that’s why they’re using it. The moment the donation stops, it ends there. We always – whenever we receive something, we always know it’s going to be – it’s always going to end at some point. It won’t be available, you understand.* [Doctor A].

Fears included post-study inability to obtain blood culture bottles, alcohol rub, and CHG solution for bathing.

## Discussion

In this study, we utilized interview methods to uncover the perceptions of doctors and nurses working in a Zambian NICU during the implementation phase of a study to evaluate a low-cost infection prevention bundle. Implementation of the bundle was successful -- rates of sepsis, BSIs and death among neonates in the NICU declined during the study period [17]. The concurrent qualitative study revealed important insights into the implementation context that can be strategically leveraged to spread and sustain the effects of the intervention. We found a high degree of enthusiasm for the bundle elements, which were described as easy to use, of low complexity and value added. Respondents identified 4 factors that facilitated the spread of infection in the NICU, including lack of a protocol for handling babies upon admission to the unit, too many admissions leading to physical crowding, an overwhelming workload that caused staff trouble in adhering to infection prevention protocols and a lack of equipment leading to overuse of devices without cleaning in between patients. Respondents suggested that NICU resource constraints reflected a societal ethos that devalued the medical care of infants. Despite the challenges, respondents were highly motivated to prevent HAIs and believed it was an achievable goal. They enthusiastically welcomed the bundle but expressed serious concern about sustainability following the study.

It has been well-established across studies of healthcare quality improvement in resource-limited settings that material deprivation, including overburdened staff, lack of equipment or supplies and insufficient funding, serves as a major barrier to the delivery of safe care [1, 4, 9, 21–23]. Our study confirms this finding while offering several novel contributions to the literature on the implementation of infection prevention in these settings.

First, by adopting a robust implementation science framework to guide analysis of our interview data, our study demonstrates the importance of considering how context may shape the implementation of infection prevention interventions. Context includes the physical, social, organizational, psychological, cultural and political dynamics surrounding a phenomenon of interest [24]. Contextual factors have been demonstrated to strongly influence the implementation of patient safety initiatives in general and infection prevention interventions in particular [10, 25]. By using CFIR to organize our findings we were able to map factors that shape HAI transmission and the implementation of the infection prevention bundle across multiple, distinct levels of influence. This is a valuable exercise because it points to areas that should be addressed in future efforts to spread and sustain the intervention.

For example, the infection prevention bundle included elements directed toward reducing contamination (CHG bathing, alcohol hand rub) and bolstering healthcare worker knowledge (text message reminders, educational workshop). This approach was successful in the short term without engaging organizational, social or political dynamics. Our findings indicate that these contextual factors may ultimately influence the long-term sustainability of the effects of the intervention. While the respondents in our study expressed a high level of motivation to engage with the bundle elements at the outset, this motivation may wane over time as contextual factors continue to make doing the right thing challenging. Strategies that focus on developing healthcare worker buy-in without attention to workload demands risk not being sustained [26]. Addressing contextual factors such as the process of admitting infants to the NICU from the L&D ward or the knowledge level of non-nursing or medical staff may improve how babies are handled to prevent infection and illustrate to NICU staff that vulnerabilities in the system that they have identified are being addressed.

Second, our findings highlight the importance of considering and modifying the spatial dimensions of the provision of care in low and middle income countries [27, 28]. While overcrowding and equipment shortages have been associated with the transmission of nosocomial infection in these settings, they have not been given much attention in the design of interventions [1, 3, 22]. Our respondents described how the physical organization of their work makes adhering to infection prevention best practices very difficult. For example, while the intervention made hand hygiene easier by making alcohol hand rub available, the location of the dispensers was seen as suboptimal given the organization of work on the unit. Patients were located so close together that the “patient zones”, or geographical areas that indicate when hand hygiene should occur, were blurred [29, 30].

The lack of visual cues to prompt hand hygiene coupled with a sense of time pressure led respondents to skip hand washing when it was indicated. While the implementation of alcohol hand rub represented a welcome change to soap and water, especially in the setting of frequent water shortages, the intervention as implemented did not go far enough. Respondents suggested a useful future modification would be personal bottles of alcohol hand rub. While this would require additional resources and coordination, it would address a commonly identified and difficult to modify spatial barrier to care. Infection prevention interventions that increase healthcare worker knowledge and provide needed resources may be bolstered by tailoring protocols to local spatial challenges and the logistics of healthcare workers moving through the physical environment.

This study has several limitations. First, the use of a qualitative approach at one hospital means that our findings may have limited generalizability. The hospital we studied, however, is unlikely to be atypical in terms of structural characteristics and challenges faced. Second, respondents may have perceived the interviewers as outsiders affiliated with the study so their answers to questions might have been biased. While this is possible, we did what we could to minimize this by clearly describing to respondents the purpose of the interviews and the confidentiality of their responses. Additionally, we did hear some critical responses and many ideas for improvement for future iterations of the intervention.

## Conclusions

This study demonstrates the value of adopting an implementation science framework to learn from those at the “sharp end” in healthcare organizations to understand the conditions that influence how care is delivered in the context of interventions to improve practice. Our findings suggest that the implementation of infection prevention interventions in NICUs located in low and middle income countries requires attention to resource limitations, the role of organizational context, spatial challenges to care delivery and post-research sustainability planning in order to bolster their impact on the safety of care delivered to vulnerable infants.

## Supplementary information

**Additional file 1.**

## Data Availability

The datasets generated and analyzed during this study are not publicly available due to the sensitive nature of the data and ethics restrictions on data sharing. Respondents did not consent to have their data publicly shared. A de-identified dataset may be available from the corresponding author on reasonable request.

## Abbreviations

BSI: Bloodstream Infection
CFIR: Consolidated Framework for Implementation Research
CHG: Chlorhexidine Gluconate
HAI: Healthcare Associated Infection
L&D: Labor and Delivery
NICU: Neonatal Intensive Care Unit
