# Supplementary material for: Healthcare worker perceptions of the implementation context surrounding an infection prevention intervention in a Zambian neonatal intensive care unit
Source: BMC Pediatr. 2020 Sep 10;20:432. doi: 10.1186/s12887-020-02323-2 (PMC7488390; doi:10.1186/s12887-020-02323-2)
Supplement: Supplementary file 1 — Additional file 1. [file 12887_2020_2323_MOESM1_ESM.pdf]

Interview Guide

Introduction/Warm Up Questions

1.) What is your job title?

2.) How long have you worked at this hospital?

3.) What is your background education and training in?

4.) What are your primary responsibilities? What types of tasks do you do each day?

5.) What are your biggest day to day stresses at work?

Nosocomial Bacteremia – General

1.) What are your thoughts about the reasons why NICU patients get bloodstream infections while getting care at this hospital?

2.) Do you think bloodstream infections in the NICU can be prevented? If yes, how? If no, can you explain why you think that?

3.) What gets in the way of preventing hospital-acquired bloodstream infections in the NICU?

4.) What helps you prevent hospital-acquired bloodstream infections in the NICU?

Nosocomial Bacteremia Prevention

Hand Hygiene

1.) How important do you think hand washing is to prevent infection in the NICU?

2.) When do you think one should wash their hands when performing patient care?

3.) What gets in the way of proper hand washing in the NICU?

4.) What helps people to wash their hands properly in the NICU?

5.) What are your opinions about the alcohol hand rub that was implemented in the NICU?

CHG Bathing

1.) How important do you think bathing patients is to prevent bloodstream infections in the NICU?

2.) What gets in the way of bathing patients in the NICU?

3.) What helps patients get bathed in the NICU?

4.) What are your opinions about the CHG bathing protocol that was implemented in the NICU?

Text Message Reminders

1.) What is your opinion about the text message/SMS reminders with infection control practices that were implemented to help reduce bloodstream infections?
    a. How well do you think they worked?
    b. Did you read them?
    c. Did you follow the advice contained within them?
    d. What do you think could be done to make them more interesting?
